# Refractory Denosumab-induced Hypocalcemia in a High-risk Patient With Osteoblastic Metastatic Prostate Adenocarcinoma

**DOI:** 10.1210/jcemcr/luaf121

**Published:** 2025-06-13

**Authors:** Jean Damascene Nizeyimana, Lisa Dreessen, Corina Andreescu, Adriaan Duhamel, Siddhartha Lieten, Eric Balti

**Affiliations:** Department of Geriatric Medicine, Universitair Ziekenhuis Brussel (UZ Brussel), Laarbeeklaan 101, 1090 Jette, Brussels, Belgium; Department of Geriatric Medicine, Universitair Ziekenhuis Brussel (UZ Brussel), Laarbeeklaan 101, 1090 Jette, Brussels, Belgium; Department of Endocrinology, Universitair Ziekenhuis Brussel (UZ Brussel), Vrije Universiteit Brussel (VUB), Laarbeeklaan 101, 1090 Jette, Brussels, Belgium; Department of Geriatric Medicine, Universitair Ziekenhuis Brussel (UZ Brussel), Laarbeeklaan 101, 1090 Jette, Brussels, Belgium; Department of Geriatric Medicine, Universitair Ziekenhuis Brussel (UZ Brussel), Laarbeeklaan 101, 1090 Jette, Brussels, Belgium; Department of Endocrinology, Universitair Ziekenhuis Brussel (UZ Brussel), Vrije Universiteit Brussel (VUB), Laarbeeklaan 101, 1090 Jette, Brussels, Belgium; Osteoporosis and Metabolic Bone Diseases Clinic, Universitair Ziekenhuis Brussel (UZ Brussel), Vrije Universiteit Brussel (VUB), Laarbeeklaan 101, 1090 Jette, Brussels, Belgium

**Keywords:** hypocalcemia, denosumab, prostate adenocarcinoma and skeletal-related events

## Abstract

Denosumab is a frequently used medication, mainly for the treatment of osteoporosis and prevention of skeletal-related events in patients with metastatic cancer. However, the treatment can be associated with adverse events including hypocalcemia.

We discuss the therapeutic challenges of denosumab-induced hypocalcemia in a patient with metastatic prostate adenocarcinoma. This 87-year-old patient presented to the emergency department after being found on the floor with altered mental status. Denosumab had been initiated 3 weeks earlier for stage 4 prostate adenocarcinoma with osteoblastic bone metastatic lesions. Blood analyses showed severe hypocalcemia (3.89 mg/dL [0.97 mmol/L]), which did not improve despite progressive incremental parenteral calcium administration and cholecalciferol supplementation. Management required 64 days of admission and titration of calcitriol. The patient was discharged after stabilizing plasma calcium level. Outpatient palliative care was later initiated because of progressive prostate adenocarcinoma, which ultimately led to the patient's death. Patients with metastatic bone disease, especially when treated with denosumab for prevention of skeletal-related events, present an increased risk of severe and even refractory hypocalcemia. More data are needed for optimal risk stratification of these patients, to identify robust predictors of hypocalcemia and to define the appropriate timing for starting calcium and vitamin D supplementation in high-risk individuals.

## Introduction

Hypocalcemia is a rare and less studied complication of malignancies, affecting approximately 10% of cancer patients [1, 2]. Those with hematological malignancies tend to be more affected [2]. In cases involving solid tumors, hypocalcemia is more commonly observed in patients with kidney failure and those with osteoblastic bone metastasis, particularly those affected by prostate cancer treated with denosumab [3-5]. Osteoblastic bone metastasis is often a significant determinant of severe hypocalcemia and requires optimal treatment of the underlying oncologic disease [4]. These patients with bone metastases are often treated with denosumab, if not a bisphosphonate, to prevent skeletal-related events (SREs) such as pathological fractures, severe bone pain, or spinal cord compression. However, denosumab, in the context of cancer treatment or not, can precipitate or exacerbate preexisting hypocalcemia [5, 6].

We describe a patient with severe refractory hypocalcemia with prostate cancer and extensive osteoblastic bone metastases who was treated with denosumab to prevent SREs. We discuss the therapeutic challenges and the potential risk factors for severe hypocalcemia while implementing treatment for prevention of SREs, with particular focus on denosumab use.

## Case Presentation

An 87-year-old man presented to the emergency department (ED) after being found on the floor in a state of altered mental status by his relatives.

He has previously undergone a right hemicolectomy for colon cancer in 2017. In 2023, an ileocolic resection was conducted for a recurrent disease at the anastomotic site of the previous surgery. This second intervention achieved optimal local disease control and distant metastases were ruled out.

**Figure 1.**
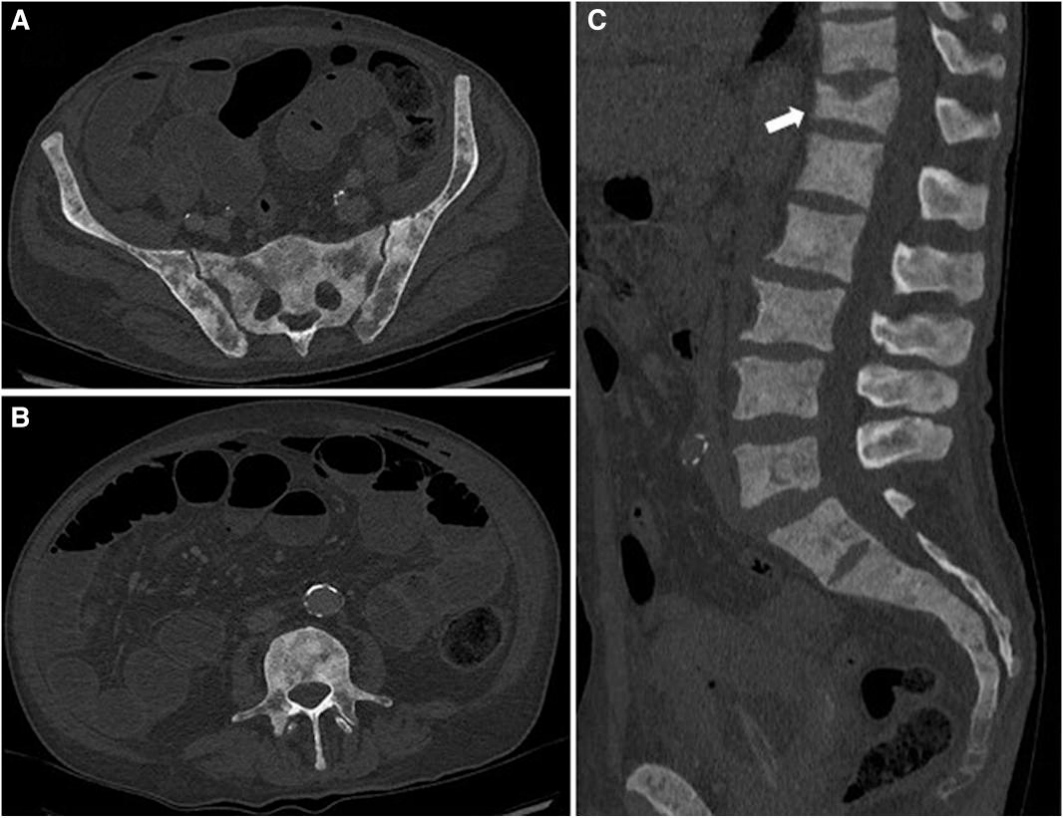
Imaging study using computed tomography scan showing diffuse osteoblastic bone lesions: (A) axial views with focus on iliac bones and sacrum; (B) axial view focused on spinal column; and (C) sagittal view showing the lumbar and dorsal columns including a T12 compression fracture (white arrow).

The patient was also known at our uro-oncology department for a metastatic stage 4 prostate adenocarcinoma Gleason 7 (3 + 4) since 2021. Hormonal castration with bicalutamide was started in 2023 and leuprorelin acetate was later added because of significant rise in prostate-specific antigen under the initial treatment. Recent computed tomography (CT) imaging showed extensive osteoblastic metastatic lesions in both femurs, humerus, the sacrum, and the vertebral column. Sacral and vertebral lesions are shown in Fig. 1. Prostatectomy was conducted and postoperative adjuvant chemotherapy was avoided due to a high Eastern Cooperative Oncology Group performance status (Eastern Cooperative Oncology Group 3 to 4) [7]. However, because of the extensive osteoblastic lesions on CT, his oncologist started denosumab 120 mg every 3 months to prevent SREs. The patient was concurrently supplemented with calcium and cholecalciferol. His first injection of denosumab was administered 3 weeks before the onset of the reported symptoms in the ED.

## Diagnostic Assessment

At the ED, the patient was somnolent, confused, and disoriented. Vital signs were within normal limits, and clinical examination was unremarkable except for a moderate cancer cachexia staging score of 50 [8].

Laboratory investigations revealed severe hypocalcemia (corrected calcium level 0.97 mmol/L [normal 2.15-2.55 mmol/L] or 3.89 mg/dL [8.62-10.22 mg/dL]), borderline low hypomagnesemia and hypophosphatemia. Intact parathyroid hormone (iPTH) was markedly elevated (440 ng/L [46.67 pmol/L]), alkaline phosphatase level was high (717 U/L [11.95 µkat/L]) and vitamin D rather low (19.2 µg/L [48.0 nmol/L]). Plasma biological parameters 1 month before admission and at the time of admission are shown in Table 1. Urinary calcium was 0.4 mg/dL/24 hours (0.1 mmol/24 hours), with a normal range of 5.21 to 15.23 mmol/L (1.3-3.8 mmol/L). The patient had been treated with calcium carbonate 1 g daily and weekly cholecalciferol 25 000 IU for a preexisting moderate hypocalcemia. Calcium level before denosumab administration is shown in Table 1.

**Table 1.** Biological parameters 1 month before, at the time of admission and 3 months after admission

Parameters	One month before admission	At the time of admission	Three months afteradmission	Normal range
Creatinine	0.87 mg/dL (76.93 µmol/L)	0.89 mg/dL (78.69 µmol/L)	0.96 mg/dL (84.88 µmol/L)	0.67-1.17 mg/dL (59.24-103.45 µmol/L)
eGFR*^a^*	78 mL/min/1.73 m^2^ (−)	77 mL/min/1.73 m^2^ (−)	71 mL/min/1.73 m^2^ (−)	>60 mL/min/1.73 m^2^ (−)
Calcium	7.49 mg/dL (1.87 mmol/L)	3.89 mg/dL (0.97 mmol/L)	9.94 mg/dL (2.48 mmol/L)	8.62-10.22 mg/dL (2.15-2.55 mmol/L)
Magnesium	2.16 mg/dL (0.89 mmol/L)	1.56 mg/dL (0.64 mmol/L)	2.04 mg/dL (0.84 mmol/L)	1.60-2.41 mg/dL (0.66-0.99 mmol/L)
Phosphate	2.48 mg/dL (0.80 mmol/L)	2.85 mg/dL (0.92 mmol/L)	3.84 mg/dL (1.24 mmol/L)	2.51-4.49 mg/dL (0.81-1.45 mmol/L)
Albumin	4.2 g/dL (42 g/L)	4.2 g/dL (42 g/L)	4.0 g/dL (40 g/L)	3.5-5.2 g/dL (35-52 g/L)
ALP	627 U/L (10.45 µkat/L)	717 U/L (11.95 µkat/L)	884 U/L (14.74 µkat/L)	40-129 U/L (0.67-2.15 µkat/L)
iPTH	na	440 ng/L (46.67 pmol/L)	17.30 ng/L (1.83 pmol/L)	15-65 ng/L (1.59-6.90 pmol/L)
Vitamin D	24.6 µg/L (61.5 nmol/L)	19.2 µg/L (48.0 nmol/L)	34.8 µg/L (87.0 nmol/L)	20-50 µg/L (50-125 nmol/L)

Abbreviations: ALP, alkaline phosphatase; eGFR, estimated glomerular filtration rate; iPTH, intact parathyroid hormone; na, not available.

^*a*^Kidney function assessed using CKD-EPI derived GFR estimate.

At the ED, an electrocardiogram showed prolonged corrected QT of 536 ms and right bundle branch block.

Follow-up thorax and abdominal CT scans showed no significant topographical lesions but the previously reported diffuse sclerotic bone metastases with radiologic evidence of osteoblastic infiltration, as well as a compression fracture of T12 as shown in Fig. 1.

## Treatment

**Figure 2.**
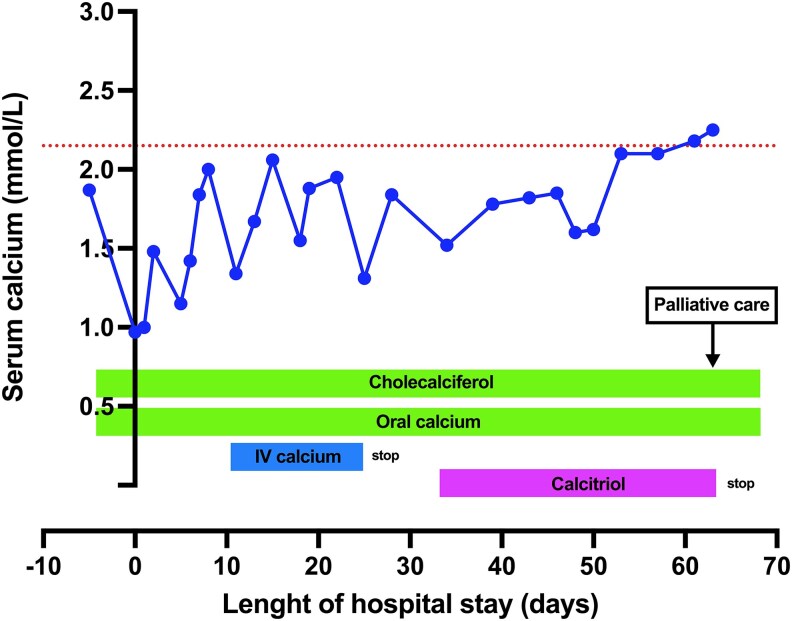
Dynamic of serum calcium level before, at the time of admission, and after admission. Timing of cessation of parental calcium supplementation and of initiation of calcitriol are indicated. The patient continuously received oral calcium and cholecalciferol. The origin of the x-axis represents the day of admission. Palliative care was initiated on discharge as illustrated. The red interrupted horizontal line indicates the lower limit of normal calcium level assessed by UV spectrophotometric method (Cobas 8000 C702, Roche Diagnostics).

The patient was initially treated with IV boluses of calcium gluconate 10% (0.225 mmol/mL elemental calcium, 1 ampule = 10 mL) and parenteral magnesium sulphate 3 g (15 mmol) daily, without improvement. Continuous IV calcium gluconate 10 g daily was then initiated in addition to oral calcium that was continued with vitamin D supplementation (Fig. 2).

## Outcome and Follow-up

Under this treatment, there was a slow and steady increase of serum calcium level, mental status improved, and QTc returned to a normal value. However, every attempt to stop IV calcium resulted to a new clinical and biological deterioration, delaying the patient’s discharge from the hospital. We concluded that the severe hypocalcemia was due to the extensive osteoblastic bone metastases in a patient with suboptimal calcium absorption capacity. This was likely precipitated by the administration of denosumab, the high burden of oncological disease (prostate adenocarcinoma), and vitamin D deficiency. This motivated the addition of calcitriol (1α,25(OH)_2_D_3_) with progressive up-titration until a steady-state plasma calcium level was achieved (Fig. 2).

On day 53 of hospitalization, plasma calcium level finally reached an asymptomatic, sustained value above 2.0 mmol/L (8.0 mg/dL). Intravenous calcium gluconate was discontinued, and the cumulative daily doses of oral calcium carbonate and calcitriol were tapered to 2.0 g and 1.5 micrograms, respectively.

The patient was discharged from the hospital after 64 days of admission. Because of progressive metastatic castration-resistant prostate adenocarcinoma, ambulant oncologic palliative care was initiated on discharge.

Three months after admission, calcium and iPTH levels remained normal and neurological findings were unremarkable. Despite this biochemical evolution, the patient died 2 months after discharge from a progressive prostate adenocarcinoma.

## Discussion

Prostate cancer is the most common cancer and the third leading cause of cancer-related death in men in Europe [9]. Approximately 10% of patients with newly diagnosed prostate cancer have bone metastases. This figure increases to 90% of patients with already known metastatic prostate cancer [10]. Disruption of calcium homeostasis is a well-known complication of bone metastases in prostate cancer, due to either osteolytic or osteoblastic lesions. Although hypercalcemia is more commonly associated with osteolytic/mixed bone disease and increased SREs, hypocalcemia is more frequent in osteoblastic calcium-avid bone lesions [4, 11]. Overall, up to 13% of patients with osteoblastic bone metastases develop hypocalcemia and in the subpopulation of patients with prostate cancer, this number rises to 27% [12].

Denosumab is a human monoclonal antibody that targets receptor activator of nuclear factor-κB (RANK)-ligand and is widely used as a bone-modifying agent to prevent or delay SREs associated with bone metastases from solid tumors, particularly prostate cancer [13]. This molecule has been reported to have at least a similar, if not better, benefit-risk ratio and adverse event profile than bisphosphonates, another class of therapies used for the same therapeutic purpose [14, 15].

Denosumab has a mean half-life of 29 days (range: 25-35 days) when administered every 4 weeks and it reaches maximum blood levels between 7 to 21 days. Peak osteoclast suppression occurs within 2 weeks following the administration [16]. Denosumab-associated hypocalcemia usually occurs on average after 1 to 2 weeks [17]. To prevent hypocalcemia following denosumab administration, prophylactic calcium and vitamin D supplementation is recommended in patients with osteoporosis and metastatic bone disease, unless the albumin-adjusted serum calcium level is high [17]. The need for vitamin D supplementation increases and becomes more important with age [18]. Identification of patients at risk of severe hypocalcemia therefore remains critical for concurrent management; ideally, this should be done before treatment with denosumab. Vitamin D deficiency, hypomagnesemia, increased oncologic disease burden (extensive metastatic bone disease), and kidney failure seem to be more associated with severe hypocalcemia during SREs prevention therapy [4, 6, 19, 20]. However, because of normal creatinine level, the potential role of kidney function in onset of hypocalcemia in our patient is uncertain. Recently, a CT radiomics-based machine learning model has been reported to be as accurate as experienced radiologists in diagnosis of osteoblastic metastatic lesions. Therefore, besides its essential recently established role in tumor grade stratification, prediction of survival, and treatment response [21], further investigation is needed to determine whether this tool could be useful for predicting incident hypocalcemia during therapies aiming at preventing SREs. Similar to the prediction of metastatic breast cancer, one could eventually assess the prediction accuracy for hypocalcemia using a composite score derived from biological and radiomics covariates [22].

**Figure 3.**
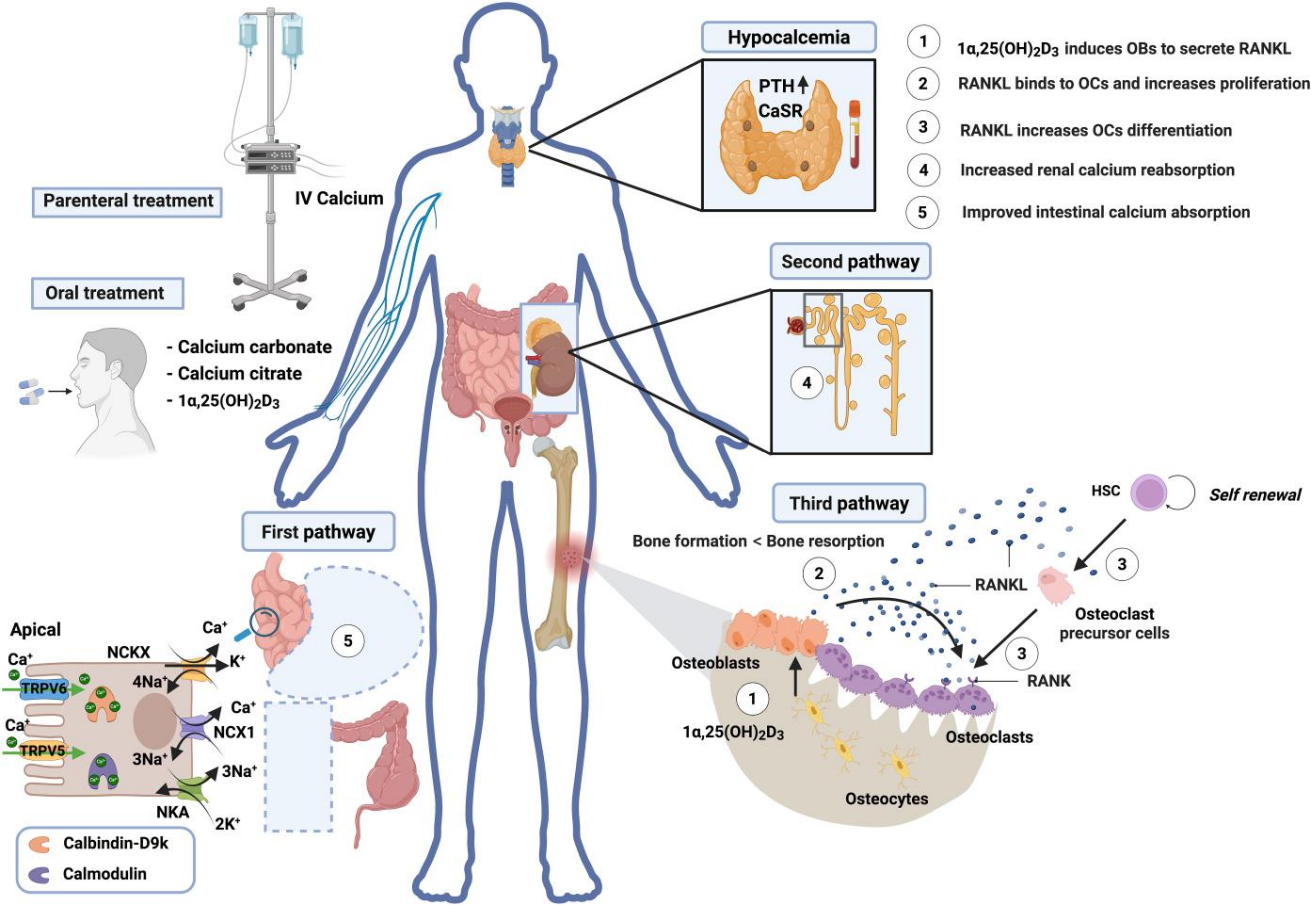
Physiologic response to maintain calcium homeostasis in hypocalcemia: elevation of iPTH due to activation of CaSR. After 25-hydroxylation in the liver and 1-alpha-hydroxylation in the kidney (not shown), calcitriol (1α,25(OH)_2_D_3_) acts on pathway 1 (intestinal calcium absorption), pathway 2 (renal calcium reabsorption), and pathway 3 (bone resorption as a result of RANKL secretion by osteoblasts that induces osteoclasts differentiation, maturation, and proliferation from HSC). In our patient, initiation of calcitriol has enabled interruption of parenteral calcium supplementation by optimizing mobilization of calcium from the skeleton, increasing renal reabsorption of calcium mainly in the proximal tubule (60%-70% of calcium absorption potency) [24], and improving intestinal calcium absorption in the remaining proximal parts of the intestine after ileo-colectomy. Calcitriol-mediated regulation of intestinal calcium absorption in this case, involves more the transcellular (instead of the paracellular) pathway by upregulating calcium-sensitive transmembrane transporters including the transient receptor potential cation channel, subfamily V, member 6 (TRPV6) and member 5 (TRPV5), K^+^-dependent Na^+^/Ca2^+^ exchanger (NCKX), Na^+^/Ca2^+^ exchanger 1 (NCX1), and Na^+^/K^+^ ATPase (NKA). The intestinal calcium homeostasis regulation process also involves calcium-binding proteins such as calbindin-D9k and calmodulin [25, 26]. Created in BioRender. Eric Balti. (2025) https://BioRender.com/bzqfv3a.

Abbreviations: CaSR, calcium sensing receptor; HSC, hematopoietic stem cell; iPTH, intact parathyroid hormone; RANKL, receptor activator of NF-κB (RANK) ligand.

In our case, the patient received denosumab with the aim of improving the quality of life by delaying the onset of pain and preventing bone fractures as well as spinal cord compression that could result from the extent of the metastases in the vertebral column and pelvis [23]. He was already at high risk of hypocalcemia before treatment due to anorexia with low-normal body mass index 19.3 kg/m^2^ (weight 57.1 kg; height 172 cm) at initiation of denosumab, calcium malabsorption previous ileocolic resection, high disease burden, a preexisting moderate hypocalcemia despite calcium supplementation, and discrete vitamin D insufficiency [20]. It is likely that mitigation of some of the risk factors, particularly hypocalcemia and vitamin D deficiency, requires a steady-state condition before initiation of RANK-targeting agents or alternative treatments to avoid/reduce the risk and/or severity of hypocalcemia. However, this hypothesis needs to be challenged in randomized clinical trials to ascertain the risk-benefit ratio of immediately initiating denosumab after calcium and vitamin D supplementation as opposed to delaying the intervention based on response to this treatment. Notwithstanding these risk factors, the concurrent osteoblastic bone infiltration likely exacerbated the hypocalcemia and could explain the sustained need of parenteral calcium supplementation. Starting and optimizing treatment with calcitriol, however, enabled stabilization of plasma calcium level allowing further treatment only with oral calcium. Our hypothesis on the actions of calcitriol on various calcium regulation pathways and homeostasis is explained in Fig. 3 [24-26].

This case highlights the need to critically select patients with metastatic bone disease, particularly those with prostate adenocarcinoma, who might benefit from treatment with denosumab to avoid hypocalcemia. This condition is not only serious but also challenging to manage. The optimal timing for initiation of RANK targeting therapy after starting calcium and vitamin D supplementation to prevent subsequent incident hypocalcemia needs further investigation.

## Learning Points

- Hypocalcemia can present as a severe and refractory metabolic abnormality in patients with prostate adenocarcinoma and metastatic bone disease undergoing treatment with denosumab for SRE prevention.
- High disease burden characterized by elevated alkaline phosphatase level or high bone-infiltrating disease and suboptimal baseline calcium level could be significant risk factors for the development of severe hypocalcemia and possibly refractory disease in patients with metastatic bone disease on SREs prevention therapies.
- There is a need to identify robust biological and/or composite predictive markers of onset of severe/refractory hypocalcemia in patients with metastatic bone disease receiving preventive therapies for SREs.

## Data Availability

Original data generated and analyzed for this case report are included in this published article.
